# A Randomized Controlled Trial of Balint Groups to Prevent Burnout Among Residents in China

**DOI:** 10.3389/fpsyt.2019.00957

**Published:** 2020-02-11

**Authors:** Lei Huang, Jennifer Harsh, Haisong Cui, Jiaxin Wu, Jessica Thai, Xu Zhang, Liming Cheng, Wenyuan Wu

**Affiliations:** ^1^ Department of Psychiatry, Tongji Hospital, Tongji University School of Medicine, Shanghai, China; ^2^ Medical Education Division, Tongji Hospital, Tongji University School of Medicine, Shanghai, China; ^3^ Department of Internal Medicine, University of Nebraska Medical Center, Omaha, NE, United States; ^4^ Tongji University School of Medicine, Shanghai, China; ^5^ College of Medicine, University of Nebraska Medical Center, Omaha, NE, United States; ^6^ Department of Orthopedics, Tongji Hospital, Tongji University School of Medicine, Shanghai, China

**Keywords:** Balint group, burnout, wellness, resident, job satisfaction

## Abstract

**Introduction:**

Burnout is highly prevalent among residents and is associated with negative outcomes for patients, organizations, and physicians. Balint groups have been shown to be an effective strategy to alleviate physicians' burnout. The purpose of this study was to examine the effectiveness and feasibility of Balint groups in preventing burnout among residents in training programs in China.

**Methods:**

36 resident physicians in their first year of residency at a comprehensive hospital in China were randomly assigned to two groups. Physicians in the intervention group participated in 2 lectures and 10 Balint sessions for 6 months, while participants in the control group were placed on a waitlist for future Balint sessions. All 36 participants completed burnout and job satisfaction measures pre and post-intervention.

**Results:**

The mean burnout subscale scores for EE and DP decreased, and the scores for PA and job satisfaction increased after Balint group participation. However, paired t-test results revealed there were no statistically significant differences between pre and post-test scores for EE (t = −1.323, *p* = 0.203), DP (t = −0.727, *p* = 0.477), PA (t = 0.716, *p* = 0.484, and job satisfaction (t = 0.282, *p* = 0.781) for the intervention group. For the control group, the burnout subscale scores for EE (t = 2.312, *p* = 0.034) and DP (t = 2.898, *p* = 0.019) increased, and there were statistically significant differences between pre and post-test. PA (t = −0.717, *p* = 0.483) and job satisfaction (t = −0.241, *p* = 0.812) scores decreased although there were no significant differences seen between the pre and post-test. Independent t-test results demonstrated statistically significant differences in scores for EE (t = −2.662, *p* = 0.013) and DP (t = −2.433, *p* = 0.020) between intervention and control group. However, there were not statistically significant differences between groups for scores in PA (t = 1.013, *p* = 0.318) and job satisfaction (t = 0.367, *p* = 0.716). All 18 participants in the intervention group reported that Balint groups were helpful and that they would be willing to attend future sessions. Overall satisfaction with the Balint group program was over 80%.

**Conclusion:**

Balint groups are an efficacious, feasible, standardized method of preventing resident burnout. Residents in China may benefit from Balint group implementation in residency training programs.

## Highlights

- A randomized controlled trial design was used to examine the effectiveness and feasibility of Balint groups in preventing burnout among Chinese residents in training programs.
- The Balint group intervention in this study was a standardized training model introduced in Germany and modified by Tongji Hospital of Tongji University.
- The Balint group participation was effective in delaying the progress of emotional exhaustion and depersonalization in Chinese residents.
- Balint groups did not significantly increase perceived personal accomplishment or job satisfaction in Chinese residents.

## Introduction

Burnout is a major concern for physicians. Researchers have reported that burnout occurs in medical trainees more often than for age-matched non-medical peers (1). A literature review revealed that burnout was prevalent for medical students (28%–45%) and residents (27%–75%, depending on specialty) as well as practicing physicians (2). A recent survey of U.S. physicians revealed that 54.4% of respondents reported at least one symptom of burnout (3). In China, the overall prevalence of physician burnout was reported as between 66.5% to 87.8% (4). Variation in reported physician burnout rate was attributed to differences in specialty, work setting, and years of training (4). From these studies, it is evident that physicians internationally are at a high risk of burnout.

Burnout rate among resident physicians, who are in the early stages of their medical careers, is high (1, 5, 6). Residency is extremely demanding with novice practitioners facing new challenges and stressors (7). Chinese residents are subject to unique occupational stressors. They have overloaded schedules, low income, feel disrespected by patients and senior physicians, and battle a growing distrust among healthcare providers and patients in China (8). Young doctors in China, especially those younger than 40 years old, have higher burnout rates than older physicians in China (4). The most recent studies on burnout rates for Chinese physicians demonstrate a range of 54.99% to 78.2% (9, 10). Studies also show that Chinese doctors facing these numerous challenges have lost their enthusiasm and regret choosing to study medicine because of low income compensation and challenging work environments (11).

Physician burnout has negative consequences not only for the physicians suffering from it but also for patients and organizations. Studies have shown that burnout can result in an increase in medical errors, reduced quality of patient care and professionalism, and decreased patient satisfaction (12–17). Within healthcare organizations, burnout is associated with lower productivity including decreased work effort, high job turnover, and early retirement (18–21). Furthermore, negative consequences of burnout for physicians in training include reductions in job satisfaction, challenges in personal relationships, substance misuse, depression, suicide, and medical illness (22–24).

Residents in China are at a high risk for burnout but few interventions exist and even fewer have been systematically tested in China specifically (25). Current findings and suggestions are not specific to Chinese doctors and lack consideration for Chinese culture. It is also unknown whether existing interventions for physician burnout can be applied to Chinese physicians (4). The purpose of this study was to determine the feasibility and effectiveness of a Balint group program to prevent burnout progression for Chinese residents *via* a randomized controlled trial.

### Physicians’ Burnout

Burnout syndrome, which was first addressed by Freudenberger (26), is a state characterized by perceptions of excessive demands, lack of enthusiasm, and feelings of frustration or cynicism due to a reduced sense of accomplishment (27, 28). Maslach's three-component model of burnout, which includes emotional exhaustion, depersonalization, and decreased sense of personal accomplishment is currently the most widely used conceptualization of burnout (29, 30).

Many factors contribute to the high risk of physician burnout, and the exact causes of burnout are complex and likely stem from a combination of both organizational and personal factors (31). Some primary drivers of physician burnout include excessive workload, imbalance between job demands and skills, a lack of workload control, prolonged work stress, work–family conflict, minimal help-seeking behaviors, and lack of support (32–36). Recently, there has been a shift from viewing burnout as an individual's problem to a problem of the health care organization as a whole, rooted in issues related to the work environment and organizational culture (37). It has been suggested that reducing physician burnout requires organizational change as well as support for physicians individually (38).

Despite burnout's serious consequences, interventions targeting physician burnout are limited; effective interventions are needed to mitigate burnout risk and impacts. Existing burnout interventions can be classified into physician-focused interventions, which target individuals, and organization-focused interventions, which target the working environment (39). The majority of previous studies have focused on physician-directed interventions and have typically involved mindfulness techniques, relaxation, stress management training, cognitive behavioral techniques, and Balint groups to reduce perceived pressure, improve communication skills, and enhance personal coping strategies (25). Organization-focused interventions include reductions in workload intensity (40), wellness teams (41, 42), and mentoring programs (43, 44). Results from the aforementioned previous studies have shown that physician-focused interventions led to very small significant reductions in burnout and further encouraged organization-focused interventions (39). Others have argued that both individual and organizational solutions are required to address burnout (45, 46).

### Balint Groups

Balint groups were developed by Michael and Enid Balint in the 1950s (47). Balint is a group training method, which aims to help physicians better understand their role in the physician–patient relationship and also assists them in improving interpersonal skills (48). Balint groups are conducted in medical training settings worldwide (49) and have been widely used as a component of medical training curricula for residents in Germany (50), Australia (51), Great Britain (52), the United States (53), and other countries internationally. Most often, these groups are conducted with family medicine trainees. In Balint sessions, participants discuss their personal experiences with patients and specifically discuss their perceptions of interactions with patients. A group leader facilitates discussion (47). The group experiences enable physicians to better handle difficult working relationships, improve empathy and communication skills, better understand their professional identity, rediscover the joy of being a physician, increase job satisfaction, and may prevent increasing levels of physician burnout (54–61).

Balint groups were introduced in China at Tongji Hospital of Tongji University as a part of the “Asia-Link project,” which focused on postgraduate training in psychosomatic medicine for physicians in China, Vietnam, and Laos. Approximately 200 Chinese physicians participated in Balint groups during the larger psychosomatic medicine training program from 2005 to 2008. Balint groups were one of the main training components of the psychosomatic medicine program, and it was highly appreciated by Chinese doctors (62, 63). Since then, Balint groups have been embedded in physician training programs in China and have become more and more popular (64).

Most Chinese studies on Balint groups have been focused on health providers' empathy and communication skills (65, 66). Very few studies have focused on the association between Balint groups and physician burnout (61, 67). To our knowledge, there have not been any previous studies that have implemented Balint groups in China with a randomized control experiment design with validated scales to assess for burnout and job satisfaction. The aim of this study was to evaluate feasibility of the Balint group program and to assess burnout and job satisfaction changes associated with Balint group participation using a randomized controlled trial study design.

## Methods

### Participants and Recruitment

All first-year resident physicians (n = 36) at Tongji Hospital of Tongji University were invited to take part in Balint groups, which were organized by the medical education division of the hospital. Twenty total available Balint group sessions were provided, of which residents were only required to attend 10 sessions. Balint group sessions were generally held every Wednesday from 17:30–18:30. The time schedule of these sessions was created based on resident availability pre-survey and was feasible for most of the residents. All residents invited voluntarily participated (100% response rate). Residents were from different specialties, including family medicine (n = 5), internal medicine (n = 2), surgery (n = 1), gynecology and obstetrics (n = 2), psychiatry (n = 2), neurology (n = 1), emergency medicine (n = 1), ophthalmology (n = 1), radiology (n = 2), and anesthesiology (n = 1). Residents were provided a snack and coffee before the Balint group. All residents were provided a certificate of completion upon completion of the required Balint group sessions.

### Measures

#### Demographic and Control Measures

Before Balint group participation, the 36 participants completed a demographic questionnaire, which included age, gender, and specialty. To control for prior experience with Balint groups, participants completed three control questions before assignment to conditions. Using a 5-point Likert response scale from 1 representing “None at all/Never” to 5 representing “Very Much,” participants were asked to rate, “How much do you know about Balint groups?”, “How many times have you participated in Balint groups?”, and “What time would you be available to attend the Balint group?” (12:00–13:00 at workday noon; 17:30–18:30 on workday; weekend).

#### Burnout

Burnout was measured using the Chinese version of the Maslach Burnout Inventory-Human Services Survey (MBI-HSS). The original MBI-HSS (68) was translated from English into Chinese and revised to make the items culturally and linguistically applicable to Chinese participants (69). The Chinese version of the MBI-HSS has previously been used with Chinese doctors and nurses (70–73). This questionnaire includes 22 items with three subscales: nine items for Emotional Exhaustion (EE), five items for Depersonalization (DP), and eight items for Personal Accomplishment (PA). The Cronbach alpha coefficients for subscales EE, DP, and PA were 0.89, 0.79, and 0.87, respectively.

#### Job Satisfaction

Job satisfaction was measured *via* the short version of the Minnesota Satisfaction Questionnaire (MSQ) developed by Weiss and Dawis (74). The Chinese version used in this study was revised by Pan (75). The 20-item short form MSQ used a 5-point Likert-type scale ranging from 1 (very dissatisfied) to 5 (very satisfied). The short form of the MSQ is scored as a composite of a number of job facets. Scores are created by summing items to illustrate each participant's satisfaction level ranging from 20 to 100. The questionnaire has been validated with Chinese health workers, and the Cronbach alpha coefficients for total job satisfaction, intrinsic job satisfaction, and extrinsic job satisfaction of the MSQ were 0.93, 0.90, and 0.83, respectively (76).

#### Satisfaction With Intervention

Satisfaction with Balint groups arrangement was measured by a questionnaire that consisted of four items including satisfaction on activity time, group size, group leader, and group atmosphere. A 5-item questionnaire was developed to assess achievements of Balint groups, where participants were asked if Balint groups were helpful in preventing increased work pressure, feeling understood and supported, improving empathy, improving communication skills, and improving occupational identity.

### Procedure

Following consent and the completion of the first round of assessment completion, the 36 residents were randomly assigned to the intervention (n = 18) or the control group (n = 18). The program started in October 2016 and ended in May 2017, lasting 6 months. Participants in the intervention group participated in a Balint group program, which included 2 lectures and 10 small group discussion sessions (at 17:30–18:30 every Wednesday). The other 18 residents in the control group were placed on the waitlist for future Balint group sessions but did not receive any intervention in the mean time. Participants in both groups completed all questionnaires, including the MBI-HSS and MSQ at the beginning of the intervention and end of the intervention time period (6 months). Participants in the intervention group were asked to submit feedback about their Balint group experience. Control group participants completed the same Balint group program form after the study was completed. Ethical clearance for the project was obtained from the Ethics Review Committee of Tongji Hospital of Tongji University (Registration Number K-KYSB-2016-100). The purpose of the study was explained to participants, and they were informed that participation was voluntary.

### Intervention

**Figure 1:**
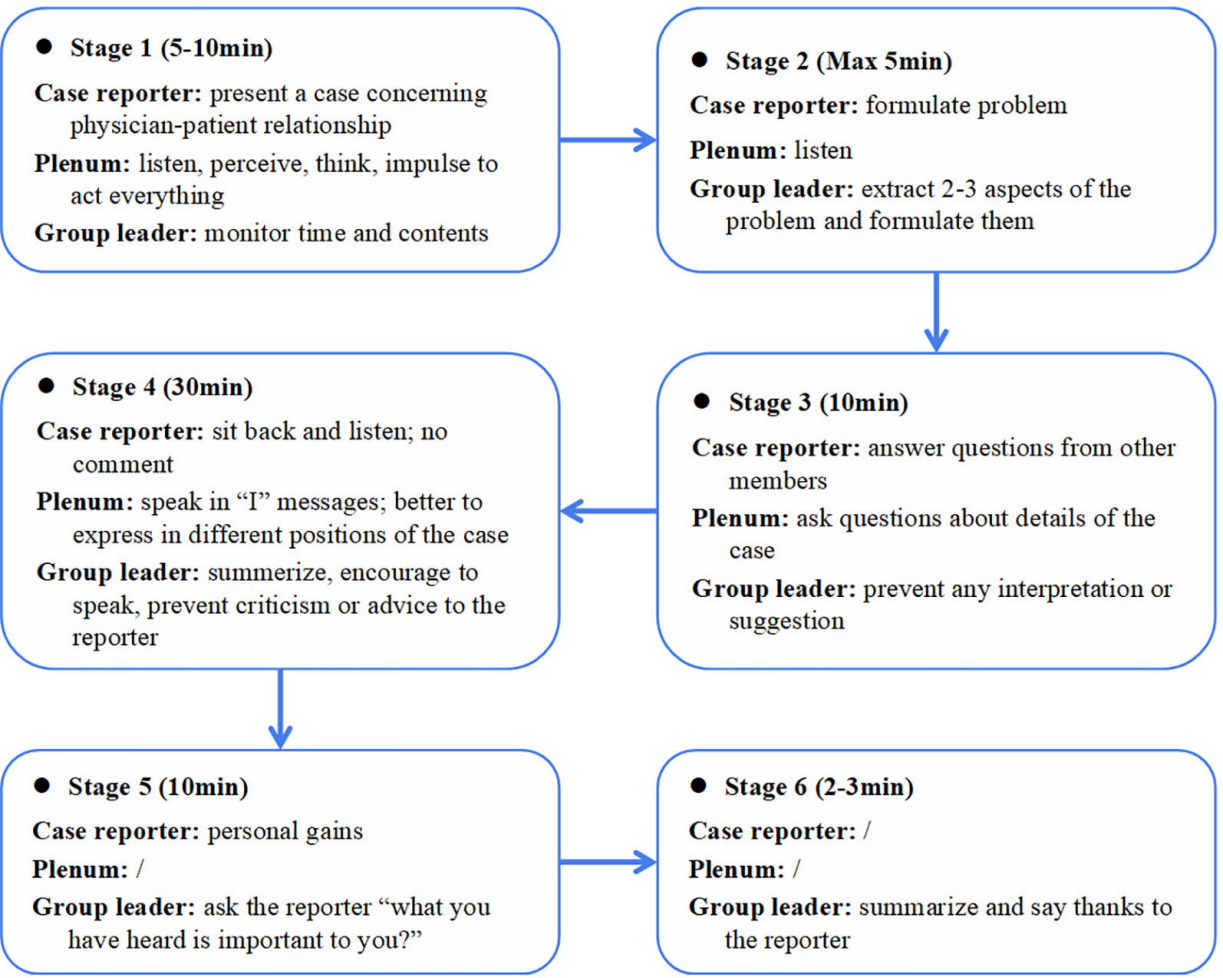
Process of a typical Balint group.

The Balint group intervention in this study was a standardized training model, which was introduced in Germany and modified by Tongji Hospital of Tongji University (77). Eight to 12 resident participants and one to two group leaders were involved in each Balint group session. Each session lasted 1 h. A volunteer before each meeting prepared a case, which showcased a challenging doctor–patient encounter. Each participant in the discussion groups could volunteer to report his/her case. The volunteer briefly described the case, then other participants and the group leader decided whether to choose the reported case as that day's topic. The group leader facilitated the entire Balint session. Discussions were largely case-focused and highlighted the emotions and attitudes aroused by participants from the presentation. Medical or technical facts were avoided. Participants were asked to consider their own reactions, emotions, and thoughts pertaining to the specific physician–patient encounter from the perspectives of both the physician and the patient. The safety of the group was attended to through clear guidelines regarding confidentiality and respect (48). **Figure 1** displays the typical process of the Balint sessions including psychological interventions such as role play and sculpture, which were used in addition to group discussion depending on what was most pertinent for each presented case.

All group leaders in this training program were formally trained and qualified by the “Asia-link Program” (62). To guarantee that residents were available for 10 sessions (taking into account their time-consuming duties, overtime work, and leave for personal reasons) 20 different Balint session options were provided based on participants' reports of the most convenient meeting times.

## Results

### Demographic Characteristics

The average age of all 36 residents was 23.67 ± 0.93. There were 11 males (30.56%) and 25 females (69.44%). There were no significant differences in age (t = −1.463, *p* = 0.153) and gender (t = 0.131, *p* = 0.717) between the intervention group and the control group. (**Tables 1** and **2**). None of the 36 (100%) residents had ever experienced Balint groups and had limited knowledge about them before the intervention. Twenty-five (69.44%) participants' preferred time for Balint group sessions was at 17:30–18:30 on workdays.

**Table 1:** Comparison of age between the intervention and the control group ($\bar{x}$ ± *s*).

Variables	Intervention group	Control group	t	*p*
Age	23.89 ± 0.90	23.44 ± 0.92	−1.463	0.153

**Table 2:** Comparison of gender between the intervention and the control group (n = 36).

Gender	Intervention group		Control group		χ²	*p*
	N	%	N	%	
Male	6	33.33	5	27.77	0.131^a^	0.717
Female	12	66.67	13	72.22				

### Effectiveness of Balint Groups on Burnout and Job Satisfaction

Independent t-tests showed that there were no significant differences for EE (t = 0.610, *p* = 0.546), DP (t = 0.402, *p* = 0.915), and PA (t = 0.186, *p* = 0.853) for burnout and job satisfaction (t = 0.402, *p* = 0.691) between the two groups before Balint groups.

The mean estimates showed that the EE and DP subscales of burnout decreased and the PA subscale and job satisfaction increased for the intervention group after Balint group participation. However, according to paired t-tests, there were no statistically significant differences between pre and post-test for EE (t = −1.323, *p* = 0.203), DP (t = −0.727, *p* = 0.477), PA (t = 0.716, *p* = 0.484), and job satisfaction (t = 0.282, *p* = 0.781). The control group's scores change for these scales showed the inverse. Paired t-tests showed that there were statistically significant differences between pre and post-test scores for EE (t = 2.312, *p* = 0.034) and DP (t = 2.898, *p* = 0.019) with no significant differences in scores for PA (t = −0.717, *p* = 0.483) and job satisfaction (t = −0.241, *p* = 0.812) for the control group (**Table 3**).

**Table 3:** Comparison of burnout subscales and job satisfaction scores between pre- and post-Balint intervention of the two groups respectively ($\bar{x}$ ± s).

Outcomes	Intervention group		t	*p*	Control group		t	*p*
	Pre-test	Post-test			Pre-test	Post-test		
Burnout subscales							
EE	16.89 ± 4.825	15.00 ± 5.111	−1.323	0.203	15.83 ± 5.533	22.17 ± 9.482	2.312	0.034
DP	7.00 ± 3.361	6.17 ± 3.130	−0.727	0.477	6.89 ± 2.847	9.72 ± 3.801	2.898	0.010
PA	28.50 ± 7.139	30.33 ± 6.843	0.716	0.484	28.06 ± 7.174	26.22 ± 6.839	−0.717	0.483
Job satisfaction	71.56 ± 5.913	72.17 ± 5.586	0.282	0.781	70.72 ± 6.524	70.11 ± 6.668	−0.241	0.812

Independent t-test results demonstrated statistically significant differences in scores for EE (t = −2.662, *p* = 0.013) and DP (t = −2.433, *p* = 0.020) between the intervention group and the control group. However, there were not statistically significant differences between groups for the score changes in PA (t = 1.013, *p* = 0.318) and job satisfaction (t = 0.367, *p* = 0.716) (**Table 4**).

**Table 4:** Comparison of burnout subscales and job satisfaction scores change between the intervention and the control group ($\bar{x}$ ± s).

Outcomes	Intervention group (post–pre)	Control group (post–pre)	t	*p*
Burnout subscales				
EE	−1.89 ± 6.057	6.33 ± 11.621	−2.662	0.013*
DP	−0.83 ± 4.866	2.83 ± 4.148	−2.433	0.020*
PA	1.83 ± 10.870	−1.83 ± 10.853	1.013	0.318
Job satisfaction	0.61 ± 9.185	−0.61 ± 10.738	0.367	0.716

*p < 0.05.

### Participants’ Satisfaction on Balint Groups

Participants in the intervention group perceived that Balint groups helped them feel understood and supported, may have prevented feeling as much perceived work pressure, improved empathy, improved communication skills, and assisted with occupation identity development (**Table 5**). Over 80% of the Balint participants were satisfied with the time and group size, while 100% were satisfied with group leaders and group atmosphere (**Table 6**). All of them were willing to attend future Balint sessions.

**Table 5:** Participants' achievements after intervention in Balint groups (n = 18).

Achievement	None at all helpful N (%)	Little helpful N (%)	Neutral N (%)	Helpful N (%)	Extremely helpful N (%)
Reduce pressure	0 (0)	0 (0)	0 (0)	6 (33.33)	12 (66.67)
Feel understood and supported	0 (0)	0 (0)	0 (0)	3 (27.27)	15 (83.33)
Improve empathy	0 (0)	0 (0)	0 (0)	6 (33.33)	12 (66.67)
Improve communication skills	0 (0)	0 (0)	0 (0)	8 (44.44)	10 (55.56)
Improve occupational identity	0 (0)	0 (0)	0 (0)	7 (38.89)	11 (61.11)

**Table 6:** Participants' satisfaction with program arrangement in the intervention group (n = 18).

Arrangement	Extremely unsatisfied N (%)	Unsatisfied N (%)	Neutral N (%)	Satisfied N (%)	Extremely satisfied N (%)
Activity time (17:30–18:30)	0 (0)	0 (0)	3 (16.67)	10 (55.56)	5 (27.78)
Group size (8–12 residents)	0 (0)	0 (0)	2 (11.11)	10 (55.56)	6 (33.33)
Group leaders	0 (0)	0 (0)	0 (0)	6 (33.33)	12 (66.67)
Group atmosphere	0 (0)	0 (0)	0 (0)	8 (44.44)	10 (55.56)

## Discussion

The aim of this study was to test whether a Balint group program could help prevent residents' burnout progression and improve their job satisfaction. We hypothesized that compared to the control group, residents participating in Balint groups would report burnout improvement. A randomized controlled trial methodology (with 36 residents) was utilized to test this hypothesis.

This study confirmed that residents who participated in a Balint group program of two lectures and 10-session small group activities reported decreases in the burnout subscales of EE and DP relative to participants whose scores increased in the control group. The comparison of scores' change for EE and DP between the groups were significantly different. However, there were not statistically significant decreases for EE and DP in the intervention group. The results indicated that Balint groups helped delay burnout progression for residents in China. This finding echoed previous study results, which demonstrated that Balint groups are an effective intervention for relieving physician burnout (57–59). These findings were also consistent with and provided much needed corroboration for a recent study among Chinese physicians conducted by Zha (61), which demonstrated that an 8-week (four sessions per participants) Balint group program decreased participants' EE and DP burnout scale and depression scores. In Zha's study, Balint groups were suggested as an effective method to prevent physicians' continued burnout under Chinese culture. However, this study did not contain a control or comparison group, the intervention time was too short, and the amount of Balint sessions were not sufficient. As such, results may have been confounded and needed to be further verified.

Balint groups focus on residents' challenges, specifically those related to physician–patient relationships, which often increase resident stress and lead to burnout (8, 78). Participating in Balint groups may be a possible way for residents to develop strategies to understand and manage difficult working relationships and challenging patient communication experiences (79). Group participation can also assist participants in recognizing and addressing their complex emotional reactions by highlighting a variety of perspectives of patient care interactions including the patients' and providers' perspectives (80, 81). Furthermore, Balint groups can assist in clarifying the physician role and normalizing challenges through shared experience (82). This can help residents increase empathy and professionalism as well as create a more positive attitude toward physician–patient relationships (60, 82, 83). Improved relationships with patients can increase physicians' sense of accomplishment and help them feel more in control of their working life (84). This, in turn, can increase resilience, enhance professional identity, may prevent emotional exhaustion and depersonalization, and improve job satisfaction, which ultimately may prevent the progression of physician burnout. These benefits are reflected in feedback from participants in the current study, who noted that Balint group participation prevented feeling as much work pressure, increased empathy, and enhanced communication skills and occupational identity.

In this study, residents spoke about their difficult encounters, clinical mistakes, and ethical problems in clinical experiences during Balint groups. This led to the most beneficial portions of the Balint group experience by highlighting residents' own frustrating experiences and emotions surrounding difficult patient encounters and helping residents learn from reflections made by other participants and group leaders. Residents were able to learn how to better manage relationships and how to recover from related stressors when their peers empathetically engaged in their distressing situations and provided support (80). Furthermore, Balint groups are intended to be a secure setting to explore and gain insight into the emotional aspects of attachment and separation of physicians from their patients (85, 86).

Results from the current study also revealed that Balint groups did not significantly increase residents' perceived personal accomplishment and job satisfaction. This was in line with results from previous studies (61, 87). These findings are not surprising since Balint groups focus on relationships and emotional reactions and do not directly focus on aspects of professionalism and well-being related to accomplishment and job satisfaction. There are additional influential factors related to personal accomplishment and job satisfaction that are complex such as income, social status, and workload (88), which Balint groups do not address. Assisting residents with these issues goes beyond Balint-type support opportunities and suggests a need for larger systems to change to create a more supportive working environment. While the Balint group interventions in this study were largely focused at the individual level, there is still a need for a larger scale, organizational change to support resident well-being in the context of these environmental stress factors.

As previously mentioned, there are few studies that evaluate outcomes of Balint group participation for physicians in China. This study adds to existing research in residency-curriculum development by using a randomized controlled experiment design and standardized assessment tools. However, our study had several limitations. First, there was a small sample size, which hindered precision, power, and generalizability and may have led to the lack of statistically significant results. Results of this study may also be confounded by the large proportion of female subjects although a randomly controlled design was used. Studies have suggested that there may be gender differences in personality traits. Females self-reported to have higher levels of neuroticism, agreeableness, warmth, and openness to feelings, and males self-reported higher levels of assertiveness and openness to ideas (89). These personality differences may have affected how participants viewed the physician–patient cases presented in the Balint groups. In addition to the limited sample size, cases presented in the Balint group sessions were based on participants volunteering to report physician–patient cases they had experienced it and a group consensus to further discuss the case. As such, there was no standardized method of determining if a case was truly suitable for discussing. Furthermore, 6 months may be a short time period to evaluate measurable changes in job satisfaction and PA. Additionally, the Balint group experience cannot be fully assessed by quantitative instruments. Including qualitative evaluation, along with quantitative, could be a useful tool to assess the Balint group experience and its impact in the future. Lastly, it may be challenging to widely implement Balint groups as group leaders require specific training, and it may be costly to staff Balint group leaders on a regular basis. Residents must also take time off from their schedules to actively and willingly participate in scheduled Balint groups. While residents in this study were very willing to accommodate to Balint group participation (participation rate of 100%), there may have been cultural influences which contributed to residents participating so willingly. Cultures have been divided by some experts into individualistic *vs*. collectivistic societies, where Asian cultures are largely classified as collectivistic. In collectivistic nations, individual autonomy is often sacrificed for obedience to authority, elders, and the greater social harmony. As such, the Chinese residents in this study may have felt compelled to participate in this study as an interpersonal obligation to their co-workers and workplace from their collectivistic upbringing. Future studies may investigate if residents in other cultures, such as Western cultures, would be as willing or receptive to Balint groups as the Chinese residents in this study (90). Group dynamics may also be unconducive to Balint groups such as if residents are especially introverted and avoid case-based discussion or are reluctant to talk to each other. Other future directions may include investigating the effects of Balint groups on each gender by holding all female or all male groups or may also investigate the effects of Balint groups among specific resident specialties as this current study included residents from a variety of fields.

## Conclusion

Balint groups are an efficacious and feasible method of preventing the progression of burnout related to EE and DP among physicians and should be implemented in residency training programs to support physician well-being in China.

## Data Availability Statement

All datasets generated for this study are included in the article/supplementary material.

## Ethics Statement

The studies involving human participants were reviewed and approved by the Ethics Review Committee of Tongji Hospital and Tongji University (Registration Number K-KYSB-2016-100). The patients/participants provided their written informed consent to participate in this study.

## Author Contributions

LH contributed to study design, recruitment of participants, data analysis and interpretation and writing of the manuscript. JH assisted in the interpretation of the results and draft writing. HC and XZ assisted in Balint Groups intervention. JW and JT assisted in recruitment of participants and draft writing. LC and WW contributed to study design and supervision. All authors have approved the final manuscript.

## Funding

This work was supported by the Priority of Shanghai Key Discipline of Medicine (2017ZZ02020); Shanghai Municipal Key Clinical Specialty (2018); Psychosomatic Medicine Project of Key Developing Disciplines of Shanghai Municipal Health Commission (2019ZB0202); Tongji University Postgraduate Educational Research and Reform Project in 2018 (2018GH33005); Key Projects of the Ministry of Education in 2019 under “The 13th Five-year Plan” for National Educational Science (DIA190409).

## Conflict of Interest

The authors declare that the research was conducted in the absence of any commercial or financial relationships that could be construed as a potential conflict of interest.

## Abbreviations

MBI-HSS, Maslach Burnout Inventory-Human Services Survey; EE, Emotional Exhaustion; DP, Depersonalization; PA, Personal Accomplishment.

## Glossary

Burnout: Refers to the chronic depletion of energy as a result of the ongoing emotional demands associated with one's occupation. It can be seen as a specific manifestation resulting from long-term, unresolvable occupational stress. Although various definitions of burnout exist, the most widely used conceptualization is Maslach's three component model, which consists of emotional exhaustion, depersonalization, and reduced feelings of personal accomplishment (91–93).
Balint groups: The concept of “Balint Group” was developed by Michael Balint as a training and research method for General Practitioners in the early 1950s. His work was first described in the book *The Doctor, His Patient and The Illness*, published in 1957 and is well known worldwide by generations of doctors. The small group is conducted under the supervision of a group leader and consists of a group of physicians who meet regularly to present clinical cases in order to improve and to better understand the physician-patient relationship (47, 94).
Job satisfaction: A feeling of fulfillment or enjoyment that a person derives from their job (95).
